# The role of *Ureaplasma in preterm* lung disease: does species matter?

**DOI:** 10.1038/s41390-026-04803-0

**Published:** 2026-01-30

**Authors:** Christine Silwedel, Christian P. Speer, Kirsten Glaser

**Affiliations:** 1 University Hospital Würzburg, University of Würzburg, Department of Pediatrics, Würzburg, Germany; 2 Division of Neonatology, Department of Women’s and Children’s Health, University of Leipzig Medical Center, Leipzig, Germany

**Commentary on** “***Ureaplasma parvum*** **and** ***Ureaplasma urealyticum*** **induce distinct types of inflammation in neonates and human epithelial cell models“ by Zhu et al**.^1^

*Ureaplasma (U.)* species (spp.) are typically considered as harmless commensals, commonly colonizing the adult urogenital tract.^2^ However, in pregnant women, strong observational and experimental evidence implicates *U. parvum and U. urealyticum* in adverse pregnancy outcomes, such as preterm premature rupture of membranes, preterm labor, and preterm birth.^2^ Furthermore, based on clinical studies and experimental work, a contributing role in neonatal morbidity, especially bronchopulmonary dysplasia (BPD), has been repeatedly discussed.^3,4^ Yet, despite considerable rates of respiratory tract colonization particularly among very preterm neonates, knowledge on the true impact of neonatal *Ureaplasma* infection and underlying mechanisms remains scarce.^3,4^

In their recent study *“Ureaplasma parvum and Ureaplasma urealyticum induce distinct types of inflammation in neonates and human epithelial cell models”*, Zhu et al. assessed *Ureaplasma*-driven inflammation in vivo and in vitro, particularly addressing potential differences in pathogenicity between the two *Ureaplasma* species.^1^ The authors analyzed tracheal aspirates from 25 intubated preterm infants <36 weeks of gestation for the presence of *Ureaplasma* spp. and inflammatory mediators.^1^ Complementary in vitro experiments assessed potential species-specific differences in the pro-inflammatory and pro-apoptotic capacity of *Ureaplasma* using immortalized alveolar epithelial cells (A549; syn. CCL-185 at the American Type Culture Collection (ATCC)) and different *U. parvum* and *U. urealyticum* serovars, both viable and heat-inactivated.

Zhu et al. found 5 out of 25 preterm infants positive for *Ureaplasma* spp., with PCR-based detection of *U. parvum* in two, and *U. urealyticum* in three patients. All *Ureaplasma*-positive infants had a gestational age below 31 completed weeks of gestation.^1^ Days on mechanical ventilation differed significantly between *Ureaplasma-positive* and -negative preterm infants (median 19 days (IQR 8.5–67.75) vs. 9.5 days (4.75–22.5)), and out of the five *Ureaplasma*-positive infants, four developed BPD.^1^ Among *Ureaplasma*-positive infants Zhu et al. found elevated levels of pro-inflammatory mediators (IL-6, IL-8, CXCL1, CXCL5, CCL20, and CCL2) in tracheal aspirates, with potentially different cytokine profiles in the *U. parvum*-positive versus the *U. urealyticum*-positive infants.^1^ In complementary in-vitro experiments, the authors described a stronger pro-inflammatory response in A549 cells incubated with *U. parvum* compared with *U. urealyticum*, whereas *U. urealyticum* seemed to trigger more cell death than *U. parvum*.^1^ These effects were observed only with viable bacteria and were absent in experiments using heat-inactivated *Ureaplasma* serovars. Both species induced oxidative stress in vitro.^1^

Based on these findings, Zhu et al. concluded that *Ureaplasma*-driven inflammation contributes to BPD development in colonized preterm infants, their study adding to the body of evidence implicating *Ureaplasma* spp. in inflammation-driven lung injury. Previous in vitro and in vivo studies have demonstrated that *Ureaplasma* infection may provoke marked pro-inflammatory cytokine and chemokine responses, accompanied by pro-apoptotic and immunomodulatory effects.^2,3,5–8^ As early as three decades ago, elevated pro-inflammatory cytokines were described in the tracheal aspirates of *Ureaplasma*-positive preterm infants.^9^ Data from animal models indicate that these inflammatory responses may result in disturbed lung development and facilitate lung injury, which may predispose to the development of BPD.^3,10^ Unfortunately, tracheal aspirate findings in this most recent study are limited by the small cohort size of only 25 preterm infants in total, including 5 *Ureaplasma*-positive neonates.^1^ In addition, the study does not specify the definition of BPD used. Also, the reported BPD incidence was exceptionally high in both *Ureaplasma*-positive and negative infants (80% and 70%, respectively), suggesting that additional BPD risk factors within the cohort warrant consideration.

Yet, *Ureaplasma* colonization in preterm neonates may progress to disease manifestation in some infants, while others present only with minor symptoms, and, to date, clinicians cannot identify infants at the highest risk of adverse outcome. Species- or serovar-specific pathogenicity might contribute to different courses and has been discussed as a critical factor promoting or modulating disease manifestation.^2,4^ Evidence, however, is scarce, and species-dependent differences in virulence have yet to be defined.

Zhu et al. attempted to close this gap of knowledge. Their results hint at potential species-specific differences in pathogenicity, suggesting that *U. parvum* predominantly drives pro-inflammatory responses, whereas *U. urealyticum* may be more likely to promote cell death—both potentially mediated by oxidative stress and secreted factors.^1^ So far, only a few in vitro studies have addressed species-dependent differences in *Ureaplasma* pathogenicity, and large-scale observational research examining this aspect within the clinical context of BPD development in preterm infants is still lacking. Some evidence points to a predominance of *U. parvum* in pregnancies complicated by preterm birth and in preterm infants later developing BPD, whereas other studies have reported no such association.^2,4^ In experiments with human pulmonary microvascular endothelial cells (HPMEC), both *U. urealyticum* and *U. parvum* were shown to induce cell death and modulate the expression of pro-apoptotic caspases; however, statistically significant effects were observed only for *U. urealyticum*.^8^ In this study, however, neither *Ureaplasma* species induced cell death in A549 cells, nor did either species trigger pro-inflammatory responses in A549 cells or HPMEC.^8^ Furthermore, a study in neonatal monocytes did not find significant differences between *U. parvum* and *U. urealyticum*-induced inflammatory responses.^6^ A comparative genome analysis of all *U. parvum* and *U. urealyticum* ATCC strains suggests that *U. urealyticum* might have a greater ability for horizontal gene acquisition, a feature discussed by the authors as possibly contributing to an increased or different virulence in certain contexts.^11^

To date, most studies have examined only single, predominantly ATCC-derived representatives of both species. Although Zhu et al. included three isolates per species, the preselection of specific serovars may still have introduced selection bias and may have influenced the observed results. Experimental studies including representative strains from all 14 serovars are highly desirable—ideally incorporating both clinical and ATCC strains. Moreover, revisiting in vitro studies of *Ureaplasma*-induced inflammation may help elucidate how different tissue or cell types vary in their immune responses to *Ureaplasma* infection, potentially contributing to differences in susceptibility or species-specific inflammatory patterns.

Insights into underlying mechanisms remain limited.^2,4^ *Ureaplasma* virulence factors have yet to be fully understood, with the multiple-banded antigen (MBA) as a surface lipoprotein family currently regarded as a potential key trigger of host immune responses.^3^ Previous work has shown that MBA size variation occurs in clinical isolates and is associated with reduced inflammation, both histologically and at the level of cytokine secretion, suggesting a role in immune evasion.^2,12^ However, based on their observation that pathogenic effects occurred only with viable pathogens, Zhu et al. discussed that soluble factors secreted by live bacteria (e.g., ammonia or phospholipases), rather than the MBA, may be the primary contributors to pathogenicity.^1^ The extent to which these descriptive data substantiate conclusions regarding underlying virulence factors warrants careful evaluation. MBA phase and size variation are well-documented in *Ureaplasma* and occur in live bacteria.

In summary, the study by Zhu et al. strengthens evidence linking *Ureaplasma* infection to inflammation-driven preterm lung injury, and highlights the importance of considering *Ureaplasma* as true pathogens in preterm infants. Moreover, the present, albeit small, study sheds light on potential differences across *Ureaplasma* species. These data add to emerging evidence for species, and potentially serovar-specific pathogenicity, and may encourage further experimental studies and future, well-powered, prospective multi-center trials evaluating the incidence of *Ureaplasma* respiratory tract colonization at the species- and, at best, at the serovar-level. So far, the picture is not yet complete.
